# Immune Thymic Profile of the MOG-Induced Experimental Autoimmune Encephalomyelitis Mouse Model

**DOI:** 10.3389/fimmu.2018.02335

**Published:** 2018-10-11

**Authors:** Sofia P. das Neves, Cláudia Serre-Miranda, Claudia Nobrega, Susana Roque, João J. Cerqueira, Margarida Correia-Neves, Fernanda Marques

**Affiliations:** ^1^Life and Health Sciences Research Institute (ICVS), School of Medicine, University of Minho, Braga, Portugal; ^2^ICVS/3B's–PT Government Associate Laboratory, Braga/Guimarães, Portugal; ^3^Clinical Academic Center-Braga, Braga, Portugal

**Keywords:** autoimmunity, multiple sclerosis, rodent, experimental autoimmune encephalomyelitis, thymus

## Abstract

Multiple sclerosis (MS) is a chronic, immune-mediated, demyelinating disease that affects the neurons of the central nervous system. Activated T cells, specific for myelin epitopes, cross the brain barriers, and react against the myelin sheath, leading to demyelination. Since T cells are generated within the thymus, here we explored, in mice, the alterations occurring in this organ throughout the different phases of the disease. We induced experimental autoimmune encephalomyelitis (EAE) in C57BL/6 females and sacrifice them at the onset (day 16) and chronic phases of disease (day 23), along with non-induced controls. We observed thymic atrophy in EAE mice at the onset that remained until the chronic phase of disease. This atrophy was associated with a preferential loss of the CD4^+^CD8^+^ double positive thymocytes, an intermediate population between the more immature CD4^−^CD8^−^ double negative and the most mature single positive thymocytes. This was accompanied by an increase in the thymic medullary/cortical ratio and by an altered expression levels of genes important for T cell survival. During the chronic phase, the thymi remained atrophic, but reacquired the normal proportion of the main four thymocyte populations and the normal medullary/cortical ratio. Importantly, at the onset phase, and accompanying these thymic alterations, EAE animals presented an increased percentage of demyelinating lesion area in the cerebellum, and an increased expression of *interferon gamma* (*Ifng*), *interleukin* (*Il) 12a*, and *Il17a*. This study suggests dynamic thymic alterations occurring in response to EAE, from the induction to the chronic phase, that might help to elucidate the MS pathophysiology.

## Introduction

Multiple sclerosis (MS) is a chronic immune-mediated demyelinating disease of the central nervous system (CNS) (1–3). Both environmental and genetic factors contribute for MS development (4, 5), but the underlying pathophysiological and etiological mechanisms are not completely understood. Currently, the animal model most widely used to study MS is the experimental autoimmune encephalomyelitis (EAE) (6–8). In the EAE model, the immunization of susceptible mice with myelin proteins induces autoimmunity against CNS components (6). Therefore, this model allows the study of several mechanisms underlying the disease, such as immune cell migration across blood-brain and blood-choroid plexus-cerebrospinal fluid barriers (9, 10) and T cell activation upon recognition of self-molecules (9). In addition, this model has been used to study mechanisms to abrogate the pathological processes involved in the development of MS, by using knockout models or testing new potential therapies (9, 11).

The thymus is a primary lymphoid organ where bone marrow derived progenitor cells undergo differentiation to originate functional T cells (12). Moreover, the thymus is also central for the control of organ-specific autoimmunity, both by limiting the output of autoreactive T cells, *via* negative selection (13), and by generating regulatory T cells (14). Concerning tolerance to self, it is known that intrathymic expression of CNS proteins, particularly by thymic epithelial cells (TECs), is important for the establishment of T-cell tolerance to self (13, 15). In the EAE model, it was shown that thymic epithelial expression of proteolipid protein (PLP) contributes to T-cell tolerance to PLP (16). Furthermore, the intrathymic administration of myelin oligodendrocyte glycoprotein (MOG)-expressing thymic epithelial progenitors induced specific tolerance against this antigen and significantly reduced EAE severity (17, 18).

In the thymus, the four main thymocyte populations are characterized by the surface expression of the co-receptor molecules CD4 and CD8. The most immature population, the CD4^−^CD8^−^ double negative (DN) cells, differentiates to CD4^+^CD8^+^ double positive (DP) cells, followed by thymocyte relocation from the cortex to the medulla, and commitment to either the CD4^+^ or CD8^+^ T cell lineage. After differentiation in the thymic medulla, CD4^+^CD8^−^ single positive (CD4SP) and CD4^−^CD8^+^ single positive (CD8SP) thymocytes leave the thymus and colonize the periphery (19). Although, the vast majority of T cells are self-tolerant, i.e., do not become activated upon encountering self-molecules, a defect in one or more of the multiple mechanisms that control self-reactivity can lead to breakdown of tolerance, ultimately resulting in T-cell mediated autoimmune diseases, as it is the case for MS (20).

Previous work has demonstrated that thymectomy in newborn mice prevented or markedly delayed the appearance of EAE symptoms (21, 22), suggesting that T cells are required for the production of signs and histological lesions. Also, MS patients were shown to present decreased thymic output (23), indicating that the thymic function could be altered in the disease. In this work, to better understand the thymic alterations occurring in response to EAE induction, we evaluated the main thymocyte populations, the thymic histological morphology and thymic gene expression in two disease time points, a more active phase (onset phase) of disease and during the chronic phase.

## Materials and methods

### Animals

All experiments were reviewed and approved by the Portuguese national authority for animal experimentation, *Direcção Geral de Veterinária* (ID: DGV9457). Animals were housed and handled in accordance with the guidelines for the care and handling of laboratory animals in the Directive 2010/63/EU of the European Parliament and the Council.

Animals were housed and maintained in a controlled environment at 22–24°C and 55% humidity, on 12 h light/dark cycles (lights on at 8 a.m.) and fed with regular rodent chow and tap water *ad libitum*.

### EAE induction and experimental groups

Disease was induced in 10–16 weeks of age female C57BL/6 mice, using a commercial kit (EK-2110; Hooke Laboratories, Lawrence, MA, USA) according to the manufacturer's instructions. Briefly, animals were immunized subcutaneously with 200 μg of MOG_35−55_, emulsified in complete Freund's adjuvant (CFA), at the upper and lower back. Pertussis toxin (PTX) in phosphate buffered saline (PBS) was administered intraperitoneally 2 and 24 h after immunization (227 ng of PTX per injection). Non-induced age-matched littermate females were used as controls and were injected subcutaneously with an emulsion of PBS in CFA (Difco Laboratories, Detroit, USA), and with PTX (List Biological Laboratories, Inc., Campbell, CA, USA) at the same concentration and time points as the EAE animals. Animals were daily weighted and monitored for clinical symptoms of disease. Disease severity was assessed daily as previously described (24) with few changes, as follows: 0 = no clinical symptoms; 0.5 = partially limp tail; 1 = paralyzed tail; 1.5 = at least one hind limb falls through consistently when the animal is placed on a wire rack; 2 = loss in coordinated movement, wobbly walk; 2.5 = dragging of hind limbs; 3 = paralysis of both hind limbs; 3.5 = hind limbs paralyzed and weakness of forelimbs; 4 = complete hind limbs paralysis and partial forelimbs paralysis; 4.5 = animal is not alert, no movement; 5 = moribund state or death. Paralyzed mice, with clinical scores above 3, were offered easier access to food and water.

### Tissue sample collection and storage

For biological sample collection, both EAE and non-induced animals were sacrificed, at the light phase of the diurnal cycle, at days 16 (onset/peak phase; maximum average clinical score, animals with scores 1.5–3.5) and 23 (chronic phase) after EAE induction. Animals were anesthetized with an intraperitoneal injection of ketamine hydrochloride (150 mg/kg, Imalgene® 1000) plus medetomidine hydrochloride (0.3 mg/kg, Dorben®). Under deep anesthesia, mice were transcardially perfused with cold 0.9% saline solution, and the thymus, brain and spinal cord were dissected. For gene expression analysis by qRT-PCR, cerebellum and thymus samples were snap-frozen and stored at −80°C. For histological analysis, the brain and spinal cord were immediately embedded in Tissue-Tek® O.C.T.^TM^ compound (Sakura Finetek, Japan), snap-frozen and kept frozen until further sectioning. 7 μm frozen thymic sections were stained with hematoxylin and eosin. For flow cytometry, thymi were placed in 3 mL of Dulbecco's Modified Eagle Medium (DMEM) with 10% fetal bovine serum and further processed.

### Flow cytometry analysis

Single-cell suspensions of thymi were prepared by mechanical dissociation, and cell suspensions were centrifuged at 1,200 rotations per minute for 10 min at 4°C. After resuspending the pellet in 2 mL of FACS buffer (0.5% bovine serum albumin, 0.01% azide in PBS buffer), the total number of cells was counted in the MUSE® cell analyzer (Merck Millipore, Darmstadt, Germany), after staining with 7-aminoactinomycin D (7-AAD) for 10 min on ice.

For flow cytometry analysis, cells were stained, for 20 min on ice, with Brilliant Violet 421 anti-mouse CD3 (clone 145-2C11; BioLegend, San Diego, CA, USA), PercpCy5.5 anti-mouse CD4 (clone RM4-5; BioLegend), and V500 anti-mouse CD8 (clone 53-6.7; BD Biosciences, Franklin Lakes, NJ, USA), washed thoroughly and resuspended in 200 μL of FACS buffer. All samples were acquired (minimum of 50000 events/sample) on an eight-color BD LSRII flow cytometer using the FACS DIVA software (BD Biosciences). Data was analyzed using the FlowJo software (Tree Star, Ashland, OR, USA) version 10.0.8. The gating strategy used is represented in Supplementary Figure 1.

### Immunofluorescence for cytokeratin (CK) 5 and CK8 in thymus sections

Serial 7 μm sections of frozen thymus were cut in the cryostat, collected to SuperFrost® Plus slides (ThermoFisher Scientific, Waltham, MA, USA), and stained with CK5 (1:500; Abcam, Cambridge, UK) and CK8 (1:500; Troma1, developed by P. Brulet and R. Kemler and obtained from the Developmental Studies Hybridoma Bank, developed under the auspices of the National Institute of Child Health and Human Development and maintained by the University of Iowa, Department of Biology, Iowa City, IA), as previously described (25). Secondary antibodies used were goat anti-rat IgG Alexa Fluor 594 (1:500; Life Technologies, ThermoFisher Scientific) and goat anti-rabbit IgG Alexa Fluor 488 (1:500; Invitrogen, ThermoFisher Scientific). Slides were visualized using a fluorescence microscope (BX61 microscope; Olympus, Hamburg, Germany) and images were posteriorly analyzed using the Fiji software (26). The total CK5 and CK8 positive areas were measured after assembling sequentially acquired images with the Fiji plugin “Stitching” (27). A total of 2 cuts per animal were analyzed, in a blind manner regarding the experimental group. Statistical analysis was performed using the average of the 2 cuts of the animal.

### Luxol fast blue staining in cerebellum and spinal cord sections

Serial 20 μm sections of frozen cerebellum and spinal cord were cut in the cryostat and collected to SuperFrost® Plus slides (ThermoFisher Scientific) and were posteriorly stained with Luxol Fast Blue. Briefly, sections were fixated in 95% ethanol at room temperature for 15 min. Next, slides were stained in 0.1% Luxol Fast Blue MBS (Chroma-Gesellshaft Schmidt GmbH&Co, Köngen, Germany) and 0.5% glacial acetic acid in 96% ethanol overnight at 56°C. The excess of dye was removed using first 96% ethanol, and then running tap water. Slides were differentiated with 0.05% lithium carbonate (Sigma-Aldrich®, St. Louis, MO, USA) for 10 min and with 70% ethanol for 1 min. After counterstaining with hematoxylin for 1 min, slides were washed with running water, left to dry on air, and mounted with Entellan® (Merck Millipore).

The quantification of the total lesioned area was done in 6–8 non-consecutive sections representative of the entire cerebellum. The sections were visualized with an Olympus microscope, and the quantification of the areas was performed with the Stereo Investigator software (MBF Bioscience, Williston, Vermont, USA), in a blind manner regarding the disease time point and group. The total white matter area was drawn using a 4x objective, and the lesion areas using the 10x objective. The percentage of lesioned area, for each section, was calculated by dividing the sum of the lesioned areas by the total white matter area. Statistical analysis was performed using the average of all sections of the animal.

### Gene expression analysis by qRT-PCR

Total RNA was extracted from the cerebellum and the thymus using TRIzol® reagent (Life Technologies) following manufacturer's instructions. RNA was quantified using the NanoDrop® 1000 spectrophotometer (ThermoFisher Scientific) and diluted to a final concentration of approximately 1 μg/μL. Next, 2-4 μg of total RNA were treated with DNase I (Life Technologies), and 500 ng of total RNA from each sample were reverse transcribed into cDNA using the iScript^TM^ cDNA synthesis kit (BioRad Laboratories, Hercules, CA, USA), according to the manufacturer's instructions. Primers used to measure the expression levels of selected mRNA transcripts by qRT-PCR were designed using the Primer-BLAST tool of the National Center for Biotechnology Information (Bethesda, MD, USA) on the basis of the respective GenBank accession numbers. All GenBank accession numbers for *Mus musculus* gene transcripts and primer DNA sequences are provided in Supplementary Table 1. The qRT-PCR was performed on a CFX96^TM^ real-time instrument (BioRad) with the SsoFast^TM^ EvaGreen® Supermix (BioRad), according to the manufacturer's instructions using equal amounts of cDNA from each sample. The cycling parameters were 1 cycle at 95°C, for 1 min, followed by 40 cycles at 95°C for 15 s, annealing temperature (primer specific) for 20 s and 72°C for 20 s, finishing with 1 cycle at 65°C to 95°C for 5 s (melting curve). Product fluorescence was detected at the end of the elongation cycle. All melting curves exhibited a single sharp peak at the expected temperature. 18S ribosomal RNA (*18S*), Adenosine Triphosphate subunit 5 beta (*Atp5b*), and Heat Shock Protein 90 alpha family class B member 1 (*Hspcb*) were used as reference genes (28).

### Statistical analysis

Statistical analysis was performed using GraphPad Prism 6.01 (GraphPad software Inc., La Jolla, CA, USA). One animal injected with MOG_35−55_/CFA emulsion was excluded from all the analysis due to absence of clinical symptoms of disease by the endpoint of the experiment. Sample normality distribution was tested using the Shapiro-Wilk (*n* > 6) and the Kolmogorov-Smirnov (*n* ≤ 6) normality tests.

For the comparison between EAE onset and EAE chronic, a two-tailed unpaired *t*-test was used, and the Cohen's d was calculated as a measure of effect size (29)—0.2, 0.5, and 0.8 were considered as small-, medium- and large-effect size, respectively (30). The non-induced, EAE onset and EAE chronic groups were compared using the parametric one-way ANOVA with Tukey's multiple comparison *post-hoc* test, for samples with normal distribution, or the non-parametric Kruskal-Wallis with Dunn's multiple comparison *post-hoc* test, for samples without normal distribution. To quantify the strength of the differences, the eta-squared value (η^2^) was calculated as a measure of effect size (29)—0.01, 0.06, and 0.14 were considered as small-, medium- and large-effect size, respectively (30). For correlations, the Pearson correlation coefficient or the Spearman's rank correlation coefficient were used for samples with normal distribution or samples without normal distribution, respectively. The strength of the correlations were quantified using the r squared.

Results are presented as mean ± standard error of mean (SEM), for parametric statistical tests, or median and interquartile range (IQR), for non-parametric statistical tests, and as the average of the experiments performed. The number of biological replicates (n) and of independent experiments are specified in the legend of each figure. Statistical significance was considered for *p* < 0.05 (^*^), *p* < 0.01 (^**^), *p* < 0.001 (^***^), *p* < 0.0001 (^****^).

## Results

### EAE induction triggered thymic atrophy

Upon EAE induction following the protocol described previously (31), animals developed a chronic disease, as expected. By day 11 post-disease induction, EAE animals started to lose weight (Supplementary Figure 2A) and to present clinical symptoms of disease (Supplementary Figure 2B). On day 16 (onset phase of disease), most animals had lost around 20% of their initial weight (Supplementary Figure 2A) and had reached an average clinical score of 3, which was maintained until day 23 (chronic phase of disease) (Supplementary Figure 2B).

**Figure 1:**
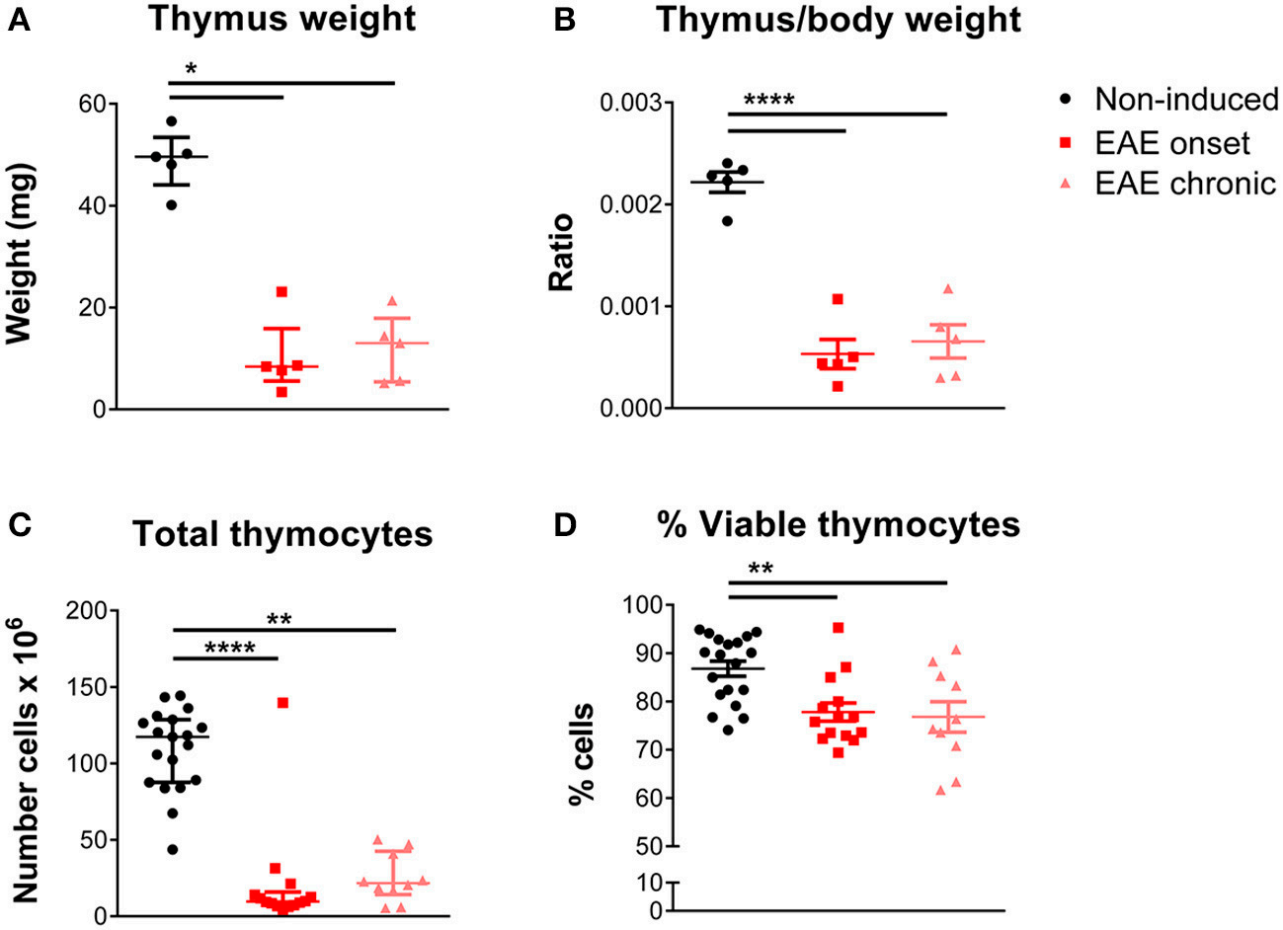
Thymic atrophy occurred after EAE induction. EAE animals, both at the onset and chronic phases, presented decreased absolute **(A)** and relative thymus weight **(B)** compared with non-induced animals (1 independent experiment; Kruskal-Wallis with Dunn's *post-hoc* test for absolute weight; one-way ANOVA with Tukey's *post-hoc* test for relative weight; *n* = 5/group). EAE animals, at the onset and chronic phases, presented a decreased total number of thymocytes **(C)** and a decreased percentage of viable thymocytes **(D)**, measured upon staining with 7-AAD (2 independent experiments; Kruskal-Wallis with Dunn's *post-hoc* test for total thymocyte number; one-way ANOVA with Tukey's *post-hoc* test for percentage of viable thymocytes; n_non−induced_ = 19, n_EAEonset_ = 14, n_EAEchronic_ = 10). Data is presented as mean ± SEM, for parametric statistical tests, or median ± IQR, for non-parametric statistical tests. *p < 0.05, **p < 0.01, ****p < 0.0001.

We observed that EAE mice presented severe thymic atrophy, at both the onset and chronic phases, as assessed by a decrease in the thymus weight [Figure 1A; $\chi_{\left(2\right)}^{2}$ = 9.42, *p* = 0.0024, η^2^ = 0.618]. This decrease was not related to the body weight loss, as it was measured after normalizing the thymic weight for the total body weight [Figure 1B; F_(2, 12)_ = 46.48, *p* < 0.0001, η^2^ = 0.886]. In agreement, the total thymocyte number [Figure 1C; $\chi_{\left(2\right)}^{2}$ = 26.66, *p* < 0.0001, η^2^ = 0.616], as well as the percentage of viable cells [Figure 1D; F_(2, 40)_ = 7.914, *p* = 0.0013, η^2^ = 0.284] were reduced in both time points.

### Thymic atrophy following EAE induction was associated with a preferential loss of the immature DP thymocytes

**Figure 2:**
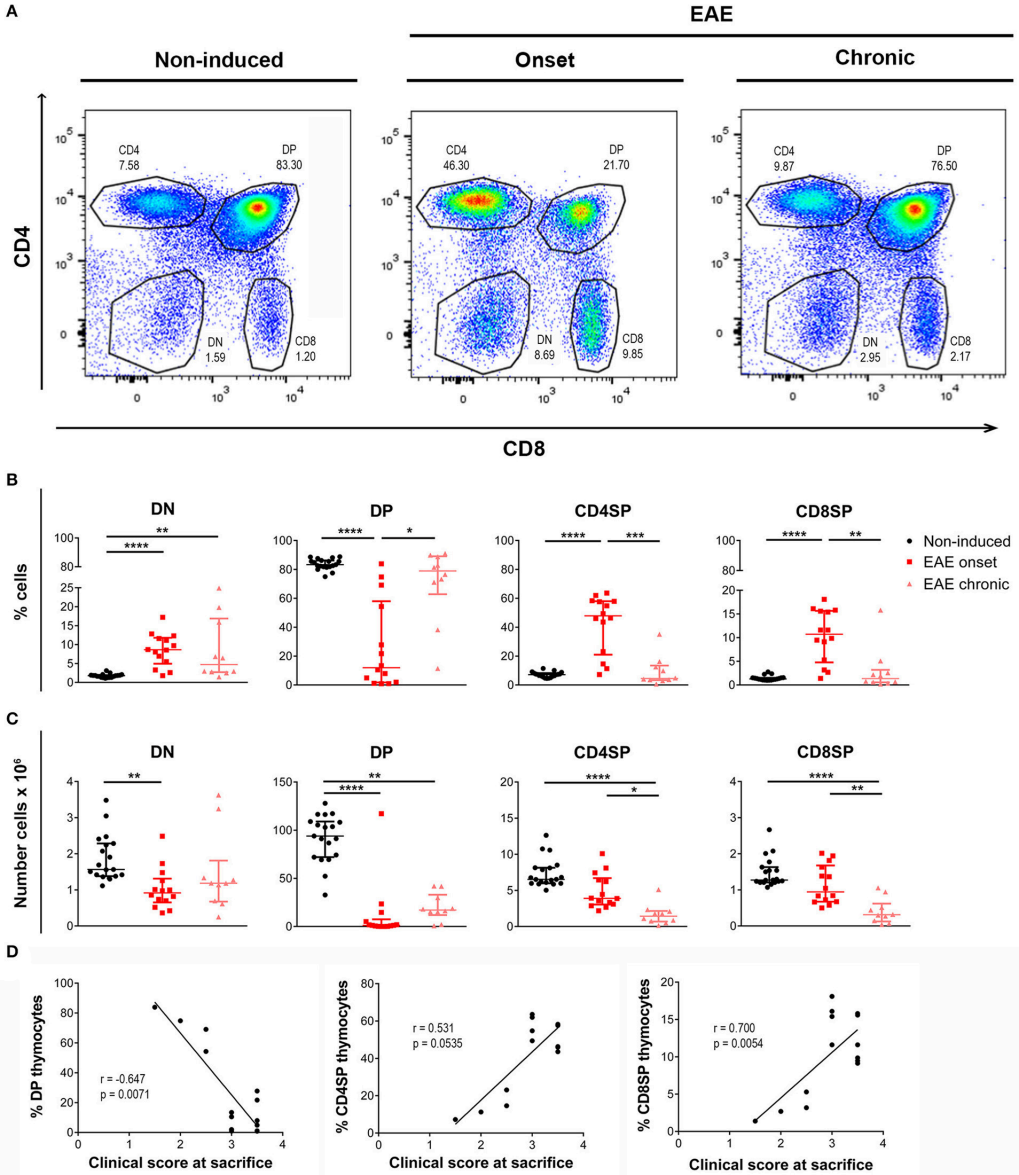
Thymocyte proportions are altered in EAE animals during the onset but not the chronic phase of disease. Flow cytometry was performed in thymic suspensions of non-induced and EAE animals, at the onset (16 days) and chronic (23 days) phases of disease, after staining with anti-CD3, anti-CD4 and anti-CD8 antibodies. Representative dot-plots of each experimental group **(A)**. During the onset phase of disease, EAE animals presented a decrease in the percentage of DP cells, and a consequent increase in the percentages of DN and SP populations (CD4SP and CD8SP); the percentages of these populations were restored to the levels of non-induced animals at the chronic phase of disease **(B)**. The absolute numbers of the evaluated thymocyte populations were decreased in EAE animals, during the onset and chronic phases of disease **(C)** (2 independent experiments; Kruskal-Wallis with Dunn's *post-hoc* test; n_non−induced_ = 19, n_EAEonset_ = 14, n_EAEchronic_ = 10). At the onset phase of disease, the percentage of DP thymocytes was negatively correlated with the clinical score of the animals on the day of sacrifice, while the percentages of CD4SP and CD8SP thymocytes were positively correlated with the clinical score **(D)** (2 independent experiments; Spearman correlation for DP *vs*. clinical score and CD4SP *vs*. clinical score, Pearson correlation for CD8SP *vs*. clinical score; *n* = 14). Data presented as median ± IQR. *p < 0.05, **p < 0.01, ***p < 0.001, ****p < 0.0001.

The proportion of the four main thymocyte populations was drastically altered at the onset, but not at the chronic phase of disease (representative CD8 vs. CD4 plots in Figure 2A). More specifically, EAE animals at the onset phase presented lower percentages of DP cells, and consequently higher percentages of DN, CD4SP, and CD8SP cells (Figure 2B). Of interest, in the chronic phase, the thymocyte populations were reestablished to the control proportions [Figure 2B; DN–$\chi_{\left(2\right)}^{2}$ = 24.12, *p* < 0.0001, η^2^ = 0.553; DP–$\chi_{\left(2\right)}^{2}$ = 21.65, *p* < 0.0001, η^2^ = 0.491; CD4SP–$\chi_{\left(2\right)}^{2}$ = 22.71, *p* < 0.0001, η^2^ = 0.518; CD8SP–$\chi_{\left(2\right)}^{2}$ = 21.17, *p* < 0.0001, η^2^ = 0.479].

Reflecting the observed decrease in total cell numbers, the absolute numbers of the main thymocyte populations were decreased at both the onset and chronic phases of disease [Figure 2C; DN–$\chi_{\left(2\right)}^{2}$ = 13.87, *p* = 0.001, η^2^ = 0.297; DP–$\chi_{\left(2\right)}^{2}$ = 27.79, *p* < 0.0001, η^2^ = 0.645; CD4SP–$\chi_{\left(2\right)}^{2}$ = 24.93, *p* < 0.0001, η^2^ = 0.573; CD8SP–$\chi_{\left(2\right)}^{2}$ = 20.93, *p* < 0.0001, η^2^ = 0.473].

Remarkably, at the onset phase of EAE, the percentage of DP thymocytes was inversely correlated with the clinical score of the animals on the day of sacrifice (Figure 2D; Spearman *r* = −0.647, *p* = 0.0071, *r*^2^ = 0.419). On the other hand, the percentages of CD4SP and CD8SP populations were directly correlated with the animals' clinical score (Figure 2D; CD4SP–Spearman r = 0.531, p = 0.0535; CD8SP–Pearson *r* = 0.700, *p* = 0.0054, *r*^2^ = 0.489).

### Altered medullary/cortical ratio and gene expression in the EAE onset

**Figure 3:**
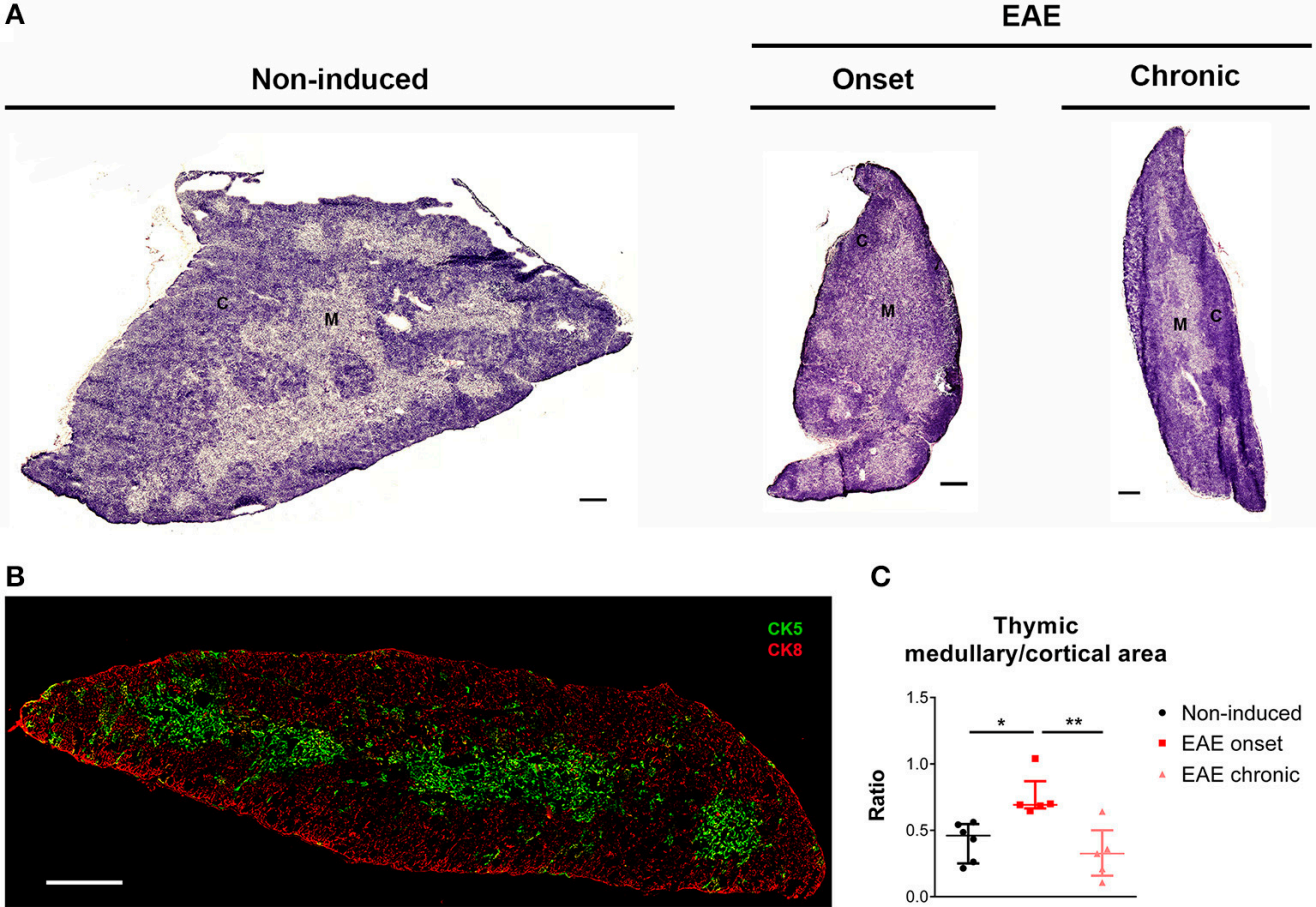
The thymic medullary/cortical ratio was altered at the onset phase of EAE. Representative thymic sections stained with hematoxylin and eosin **(A)** (scale bar indicates 300 μm). Thymic section evidencing medullary epithelial cells in green (CK5+) and cortical epithelial cells in red (CK8+) **(B)** (scale bar indicates 300 μm). The medullary/cortical ratio was increased in EAE animals during the onset, but not the chronic phase of disease **(C)** (1 independent experiment; Kruskal-Wallis with Dunn's *post-hoc* test; n_non−induced_ = 6, n_EAEonset_ = 5, n_EAEchronic_ = 5). Data presented as median ± IQR. *p < 0.05, **p < 0.01. C, cortical layer; M, medullary layer.

To investigate to what extent the thymic structure was also affected during EAE, we performed an hematoxylin and eosin histochemical staining (Figure 3A). We confirmed, at the histological level, that EAE animals presented thymic atrophy, and it was possible to observe a decrease in the cortical layer at the onset phase of disease. To confirm this, we next performed a double staining for CK5 and CK8, which are preferentially expressed in thymic epithelial medullary and cortical cells, respectively (Figure 3B), and quantified the ratio between the medullary and cortical layers. As expected, since DP thymocytes are mostly present in the cortex and the proportion of these cells is severely affected in the onset, we observed an increase in the medullary/cortical ratio at the onset, but not at the chronic phase of EAE [Figure 3C; $\chi_{\left(2\right)}^{2}$ = 10.11, *p* = 0.0012, η^2^ = 0.624].

**Figure 4:**
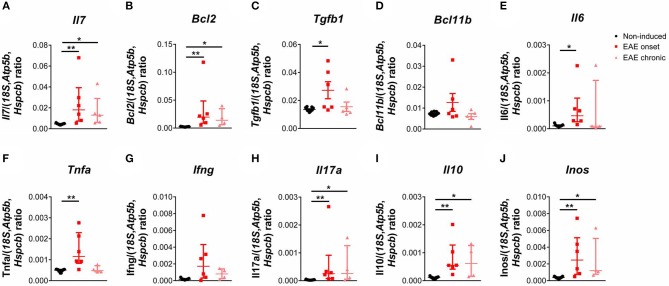
The expression levels of pro-inflammatory cytokines and of genes important for DP thymocyte differentiation and survival were altered during EAE. The expression levels of *Il7* **(A)**, *Bcl2* **(B)** and *Tgfb1* **(C)** were increased in the thymus of EAE animals, while *Bcl11b* was not altered **(D)**. The thymic expression levels of *Il6* **(E)**, *Tnfa* **(F)**, the pro-inflammatory cytokines *Ifng* **(G)** and *Il17a* **(H)**, the anti-inflammatory cytokine *Il10* **(I)** and *Inos* **(J)** were also increased in EAE animals (1 independent experiment; Kruskal-Wallis with Dunn's *post-hoc* test for *Il7, Bcl2, Ifng, Il17a, Il10, Il6, Inos* and *Tnfa* one-way ANOVA with Tukey's *post-hoc* test for *Bcl11b* and *Tgfb1*; n_non−induced_ = 11, n_EAEonset_ = 6, n_EAEchronic_ = 4 for *Bcl2, Ifng, Il17a, Il10, Il6, Inos* and *Tnfa*, n_non−induced_ = 11, n_EAEonset_ = 6, n_EAEchronic_ = 5 for *Il7, Tgfb1* and *Bcl11b*). Data presented as mean ± SEM, for parametric statistical tests, or median ± IQR, for non-parametric statistical tests. *p < 0.05, **p < 0.01.

We next quantified the expression levels of genes important for thymocyte differentiation and survival, and also of genes relevant in the EAE context. We found a significant increase in the expression levels of *interleukin* (*Il) 7* in EAE animals [Figure 4A; $\chi_{\left(2\right)}^{2}$ = 14.85, *p* < 0.0001, η^2^ = 0.676]. IL-7 signaling promotes cell survival by inducing expression of anti-apoptotic genes such as B-cell lymphoma 2 (*Bcl2*), which we also found increased in the thymus of EAE animals [Figure 4B; $\chi_{\left(2\right)}^{2}$ = 15.10, *p* < 0.0001, η^2^ = 0.728]. Moreover, we observed that the expression levels of transforming growth factor beta 1 (*Tgfb1*), which presents an anti-apoptotic role in thymocytes, were increased in the onset phase [Figure 4C; F_(2, 19)_ = 5.36, *p* = 0.0142, η^2^ = 0.361]. B-cell lymphoma/leukemia 11B (*Bcl11b*), which is involved in the positive selection and survival of DP thymocytes (32), was not altered in EAE animals [Figure 4D; F_(2, 19)_ = 2.53, *p* = 0.1063]. IL-6 and tumor necrosis factor alpha (TNFa) were previously associated with thymic atrophy, and their gene expression was increased in EAE animals at the onset phase of disease [Figures 4E,F; *Il6*–$\chi_{\left(2\right)}^{2}$ = 8.75, *p* = 0.0064, η^2^ = 0.375; *Tnfa*–$\chi_{\left(2\right)}^{2}$ = 10.20, *p* = 0.0019, η^2^ = 0.456]. In addition, the thymic expression of pro-inflammatory cytokines relevant for the disease such as *interferon-gamma* (*Ifng)* [Figure 4G; $\chi_{\left(2\right)}^{2}$ = 7.18, *p* = 0.0200, η^2^ = 0.288] and *Il17a* [Figure 4H; $\chi_{\left(2\right)}^{2}$ = 13.44, *p* < 0.0001, η^2^ = 0.636] were increased in EAE animals, compared to non-induced animals. The anti-inflammatory cytokine *Il10* presented increased expression levels at the onset and chronic phases of disease [Figure 4I; $\chi_{\left(2\right)}^{2}$ = 14.53, *p* < 0.0001, η^2^ = 0.696]. Inducible nitric oxide synthase (*Inos*) was also found up-regulated in EAE animals in both disease time points [Figure 4J; $\chi_{\left(2\right)}^{2}$ = 13.94, *p* < 0.0001, η^2^ = 0.663]. For the majority of genes quantified, the increase observed at the chronic phase was less pronounced than the one observed at the onset phase.

### The cerebellum exhibited a different inflammatory response in the EAE onset and chronic phases

**Figure 5:**
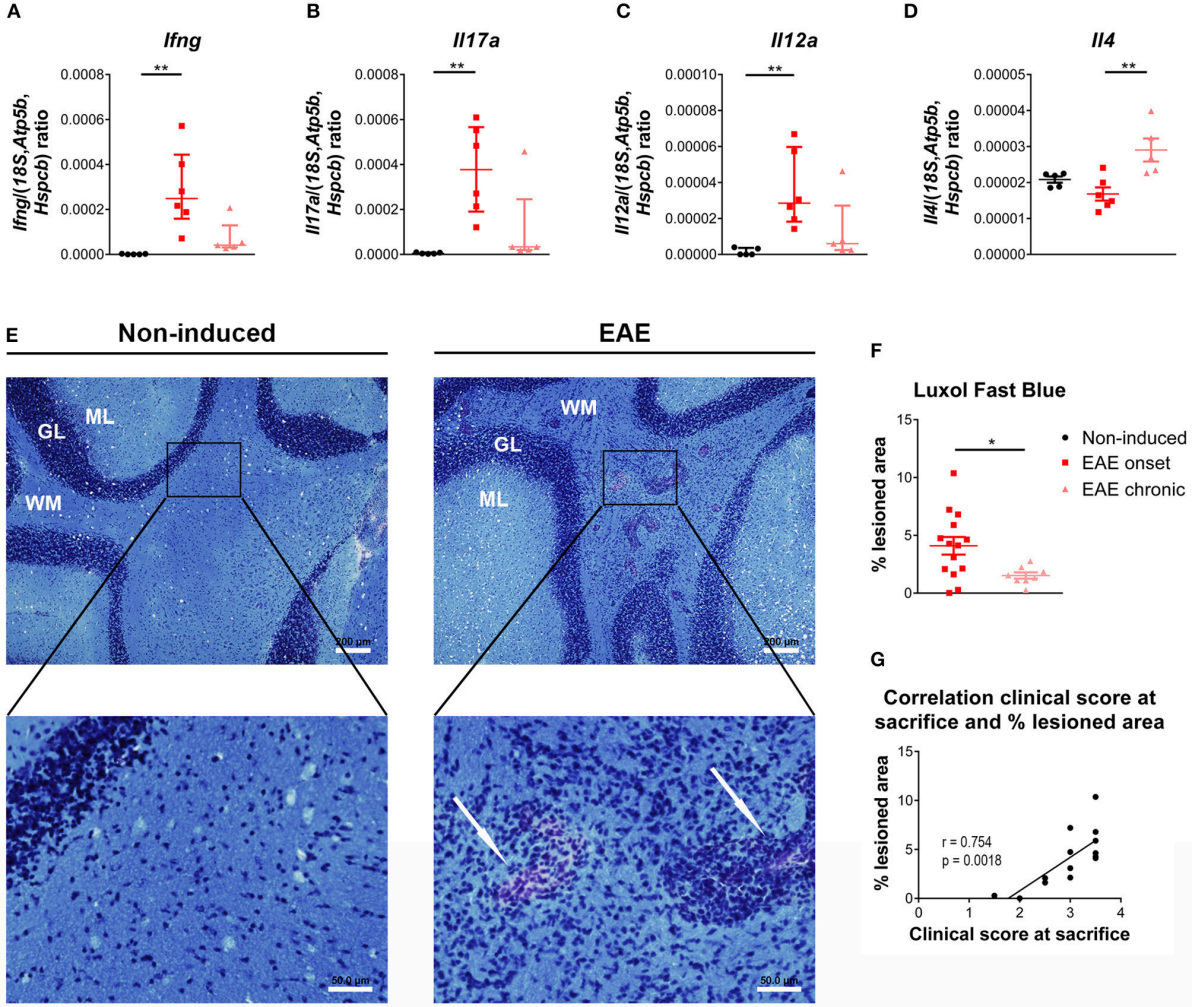
Distinct inflammatory profile in the cerebellum at the onset and chronic phases of disease. At the onset phase of disease, EAE animals presented an increased expression of the pro-inflammatory cytokines *Ifng* **(A)**, *Il17a* **(B)** and *Il12a* **(C)**, compared to non-induced animals. The expression levels of the Th2 cytokine *Il4* were altered in chronic EAE animals compared to the onset phase of disease **(D)** (1 independent experiment; one-way ANOVA with Tukey's *post-hoc* test for *Il4*, Kruskal-Wallis with Dunn's *post-hoc* test for remaining genes; n_non−induced_ = 5, n_EAEonset_ = 6, n_EAEchronic_ = 5). The Luxol Fast Blue staining evidenced lesion areas, characterized by inflammatory infiltrates and lighter blue staining, in the cerebellum' white matter of EAE animals (arrows, **E** right bottom panel), which were not present in non-induced animals (**E** left panel) (scale bars indicate 200 μm for lower magnification, and 50 μm for higher magnification). EAE animals presented a significant increase in the percentage of lesioned area at the onset phase of disease, compared with the chronic phase **(F)** (2 independent experiments; unpaired *t* test; n_EAEonset_ = 14, n_EAEchronic_ = 8). At the onset phase of disease the percentage of lesioned area correlated positively with the clinical score **(G)** (2 independent experiments; Pearson correlation, *n* = 14). Data presented as mean ± SEM, for parametric statistical tests, or median ± IQR, for non-parametric statistical tests. *p < 0.05, **p < 0.01. GL, granular layer; ML, molecular layer; WM, white matter.

Previous studies have shown that the cerebellum is affected in EAE (33–35), therefore, and to find correlates with the peripheric thymic alterations, we investigated the expression of T-cell associated cytokines in this region. At the onset phase, we observed an increase in the expression levels of pro-inflammatory cytokines associated with Th1 and Th17 cells, respectively *Ifng* and *Il17a* [Figures 5A,B; *Ifng*–$\chi_{\left(2\right)}^{2}$ = 12.51, *p* < 0.0001, η^2^ = 0.808; *Il17a*–$\chi_{\left(2\right)}^{2}$ = 12.04, *p* < 0.0001, η^2^ = 0.772]. In addition, *Il12a*, involved in the induction of a Th1 response, was also increased at the onset phase [Figure 5C; $\chi_{\left(2\right)}^{2}$ = 9.96, *p* = 0.0016, η^2^ = 0.612]. At the chronic phase of disease, the expression levels of these cytokines returned to control levels (Figures 5A–C). *Il4* expression, a cytokine associated with Th2 cells, was increased at the chronic phase of EAE, compared to the onset phase [Figure 5D; F_(2, 13)_ = 8.34, *p* = 0.0047, η^2^ = 0.562].

After staining with Luxol Fast Blue (Figure 5E), lesion areas in the white matter were identified by a paler blue staining (or absence of staining) and by the presence of inflammatory infiltrates (arrows on right bottom panel of Figure 5E). The total area occupied by these lesions was quantified, and at day 16 post-EAE induction, known to be the most active/severe phase of disease, animals displayed a significant increase in total lesioned area when compared with the chronic phase (day 23 post-EAE induction) [Figure 5F; t_(20)_ = 2.461, *p* = 0.0231, *d* = 1.224]. Moreover, during the onset phase, the percentage of lesioned area was positively correlated with the clinical score of animals [Figure 5G; Pearson *r* = 0.754; *p* = 0.0018; *r*^2^ = 0.568]. EAE animals also presented lesioned areas in the spinal cord' white matter (Supplementary Figure 3).

## Discussion

Herein, we analyzed, in the EAE chronic model, the thymic alterations in two important disease time points—onset and chronic phases. In both, we observed thymic atrophy, assessed by decreased thymic weight and total cell numbers. However, the pattern of thymic atrophy was distinct in the two EAE phases: while at the onset the percentage of DPs is extremely reduced, in the chronic phase all thymocyte populations are equally reduced in number and the proportions of the four main populations is re-established. Notably, these alterations in the thymus were associated with inflammation in the CNS. Namely in the cerebellum, at the onset phase, animals presented an increased percentage of lesion sites and expression levels of pro-inflammatory cytokines, which was no longer observed at the chronic phase. Moreover, in the cerebellum we found that *Il4* expression was increased at the chronic phase. Of relevance, the intrathecal injection of an IL-4-expressing viral vector, after EAE induction, was shown to significantly decrease disease severity, reduce demyelination and axonal damage and also to increase the percentage and absolute numbers of Foxp3+ Treg cells, in the CNS (36). In accordance, the increase in *Il4* expression, in the cerebellum, might represent a recovery mechanism possibly associated with a decrease in the local pro-inflammatory response and increased recruitment of Treg cells. Also, in EAE animals, it was shown a gradual increase in the thymic production of Treg cells from the onset to the chronic phase (37).

Of interest, altered thymic activity had already been suggested in relapse-remitting MS patients (23) and thymic atrophy was also found in other MS models, namely the cuprizone model (38), in A.SW mice with primary-progressive EAE (39) and also, recently, in rat EAE models (40). Herein we also showed thymic atrophy accompanied by decreased percentage of viable cells, that could result from apoptotic cell death, as observed in the rat EAE models (40).

Here we found that, regarding the main thymocyte populations, the onset phase was associated with a 70% decrease of DP thymocyte percentage and a 90% of total DP thymocytes, compared to non-induced animals. In accordance, a previous study described a similar decrease of DP thymocytes in an EAE model induced with recombinant MOG, at day 18 post-disease induction (39). In addition, we also show here that at the chronic phase, the number of CD4SP and CD8SP thymocytes was even further decreased and the total number of DP thymocytes increased by 40%, when compared to the onset phase, contributing for the normalization of the thymocyte proportions.

Distinct mechanisms underlying the observed thymic atrophy may occur. Depending on their levels, glucocorticoids were found to play both positive and negative effects on thymocytes—low levels are associated with thymocyte survival and proliferation, and high levels induce thymocyte apoptosis (41). Moreover, glucocorticoids present immunosuppressive properties, and have been used in the treatment of several inflammatory and autoimmune pathologies, including MS (42). Nevertheless, elevated concentrations of glucocorticoids were shown to trigger severe thymic atrophy, mainly by inducing DP thymocytes apoptosis (43). Of interest, a previous study has found a progressive decrease in thymic weight with EAE progression, accompanied by increased corticosterone levels (44). Furthermore, increased levels of pro-inflammatory cytokines, including IL-6, were shown to induce acute thymic atrophy *via* loss of DP thymocytes (45, 46). In fact, we observed an increased thymic expression of *Il6, Ifng*, and *Il17a*, in EAE animals, and this increase was more pronounced at the onset phase of disease. These possible mechanisms could be contributing to the thymic atrophy and loss of DP thymocytes observed in this study.

Regarding thymic histology, at the onset phase of disease, the cortical layer presented increased atrophy when compared to the medullary layer. This is consistent with the observed decrease in the DP population, which is the most abundant population in the cortical layer. On the other hand, at the chronic phase, accompanying the restitution of the thymocyte populations' percentages, the medullary/cortical ratio was also restored to control levels.

In terms of thymic gene expression, we found alterations in genes related with thymocyte survival and differentiation. IL-7 is produced by thymic stromal cells and plays a critical role in thymocyte survival, by inducing the expression of anti-apoptotic factors like *Bcl2* (47, 48), and in the maturation of DN cells (49). Similarly to IL-7, TGFb1 was shown to play an anti-apoptotic role in thymocytes (50), by enhancing *Bcl2* expression levels and reducing the pro-apoptotic *Bim* levels (51). All these genes were found to be up-regulated during the onset phase of EAE, suggesting a compensatory mechanism to increase the survival and differentiation of immature thymocytes, promote the transition to DP thymocytes and ultimately lead to thymic recovery. In fact, at a later disease time point, the chronic phase, the expression levels of these cytokines were decreased, compared to the onset phase, and, although the thymic atrophy persisted, the percentage of the DP thymocytes was normalized.

Altogether, we describe here that the thymus presents dynamic alterations throughout EAE development. Other studies have also shown thymic atrophy in EAE animals, however here we describe that thymocyte proportions are altered in a more active phase of disease, the onset, but not during the chronic phase. In addition, the medullary-cortical ratio is clearly altered, but only at the EAE onset. In the future, the modulation of the corticosterone levels and of the thymic response could help to elucidate if the thymic alterations are solely a consequence of increased corticosterone and/or increased production of pro-inflammatory cytokines, or if there is a cause-consequence relation between what happens in the CNS and in the thymus. Importantly, understanding the thymic alterations in the MS context may help to develop new therapeutic strategies, focused on the thymus, and that may be able to modulate disease progression.

## Author contributions

SdN and CS-M performed the experiments; SdN performed the data analysis and wrote the manuscript; CN, SR, and JC critically revised the manuscript; MC-N was involved in revising the manuscript critically for important intellectual content and made substantial contributions to the interpretation of data; FM designed and supervised the study and edited the manuscript. All authors read and approved the final manuscript.

### Conflict of interest statement

The authors declare that the research was conducted in the absence of any commercial or financial relationships that could be construed as a potential conflict of interest.
