# Supplementary material for: Immune Thymic Profile of the MOG-Induced Experimental Autoimmune Encephalomyelitis Mouse Model
Source: Front Immunol. 2018 Oct 11;9:2335. doi: 10.3389/fimmu.2018.02335 (PMC6194318; doi:10.3389/fimmu.2018.02335)
Supplement: Supplementary file 1 [file Data_Sheet_1.docx]

Supplementary Material

**Immune thymic profile of the MOG-induced experimental autoimmune encephalomyelitis mouse model**

**Sofia P. das Neves, Cláudia Serre-Miranda, Claudia Nobrega, Susana Roque, João J. Cerqueira, Margarida Correia-Neves, Fernanda Marques^*^**

^*^ **Correspondence**: Corresponding Author: fmarques@med.uminho.pt

**
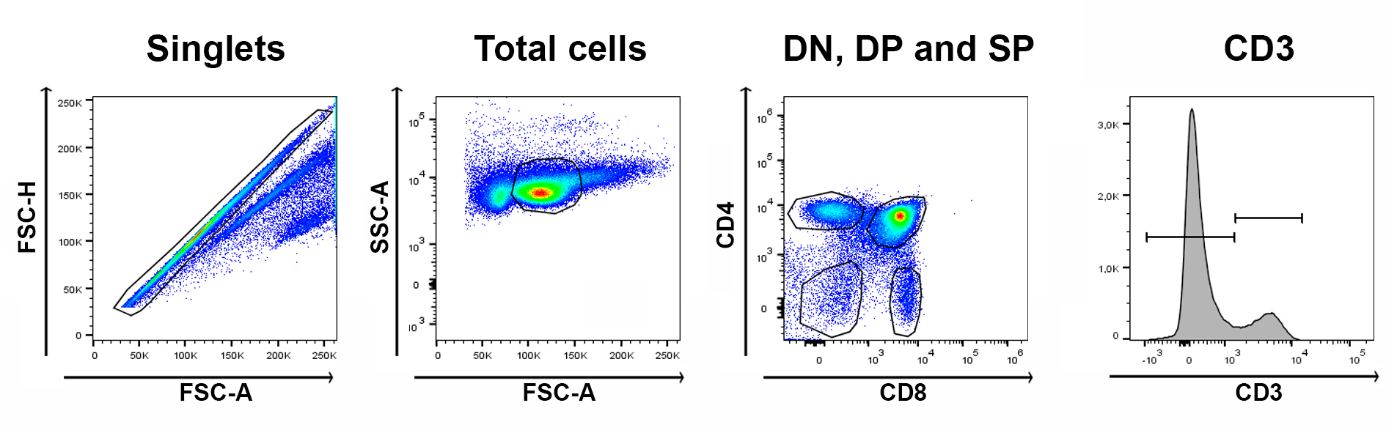
**

Supplementary Figure 1 - Gating strategy used to analyze the flow cytometry data. Singlets were gated using the FSC-A *vs*. FSC-H plot, followed by exclusion of cell debris using the FSC-A *vs*. SSC-A plot. The DN, DP, CD4SP and CD8SP populations were gated in the CD8 *vs*. CD4 plot, within total cells. The presence of CD3 expression was evaluated by histogram, for CD4SP and CD8SP populations.


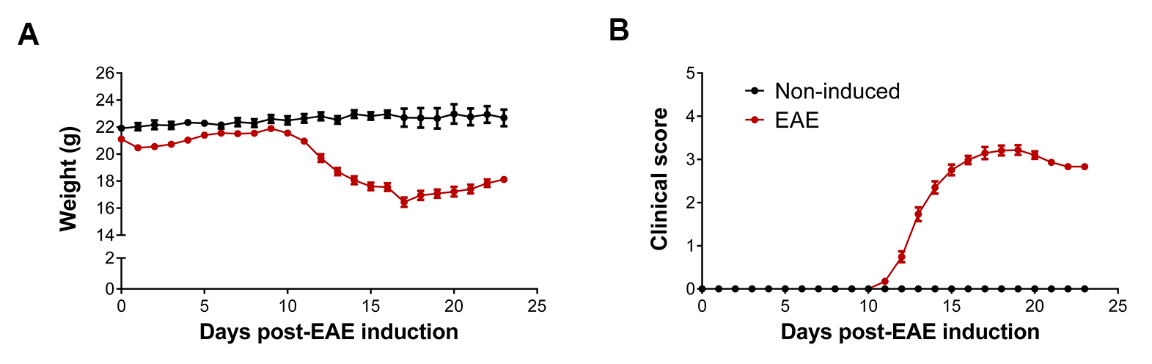


Supplementary Figure 2 – Disease progression of a chronic model of EAE in C57BL/6 female mice. C57BL/6 females were injected with an emulsion containing MOG_35-55_ to induce EAE and were weighted and monitored daily for clinical symptoms of disease. Non-induced mice were used as controls. EAE mice started losing weight by day 11 post-EAE induction (A), when they also started to present the first clinical symptoms of disease (B) (2 independent experiments; n_non-induced_ = 30, n_EAE_ = 37). By day 16, animals had already lost around 20% of their initial weight and the disease reached a peak of activity, and from this day until the end of the experiment, animals maintained similar weights and clinical scores. Both experimental groups, EAE and non-induced, were sacrificed on days 16 (onset) and 23 (chronic) post induction. Data presented as mean ± SEM.


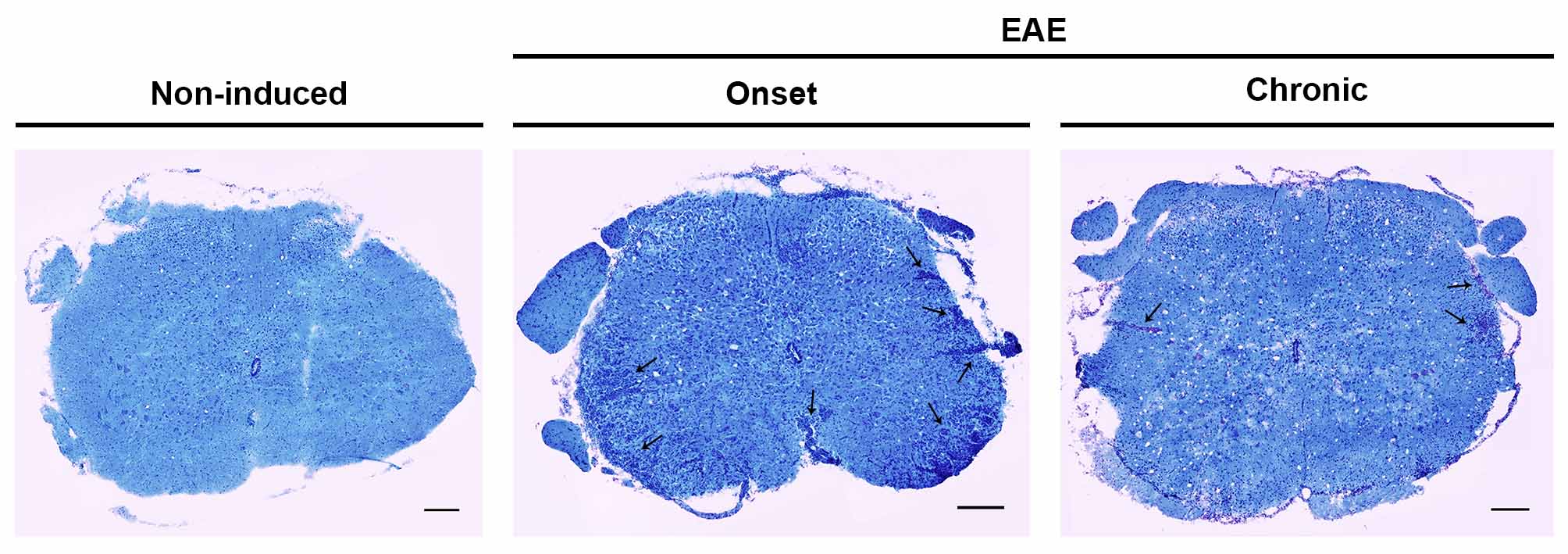


Supplementary Figure 3 – EAE animals presented lesioned area in the spinal cord’ white matter. After staining spinal cord sections with Luxol Fast Blue it was possible to observe lesioned areas in the white matter of EAE animals, presenting inflammatory infiltrates and paler blue staining (arrows center and right panel), which were not present in non-induced animals (left panel) (scale bars indicate 200 µm).

Supplementary Table 1 – Genes analyzed by qRT-PCR, respective primers sequences and GenBank accession numbers.

| Gene symbol | Primer sequence  (5’ → 3’) | Annealing temperature (ºC) | GenBank accession number |
| --- | --- | --- | --- |
| *18S* | Forward: CGGCTACCACATCCAAGGAA | 59 | NR_003278.3 |
|  | Reversal: GCTGGAATTACCGCGGCT |  |  |
| *Atp5b* | Forward: GGCCAAGATGTCCTGCTGTT | 60 | NM_016774.3 |
|  | Reversal: GCTGGTAGCCTACAGCAGAAGG |  |  |
| *Bcl11b* | Forward: TACTGTCACCCACGAAAGGC | 60 | NM_001079883.1 |
|  | Reversal: TGGGAAGAGGAGGCAGCTAT |  |  |
| *Bcl2* | Forward: GGATGCCTTTGTGGAACTG | 57 | NM_009741.5 |
|  | Reversal: CAGCCAGGAGAAATCAAACAG |  |  |
| *Hspcb* | Forward: GCTGGCTGAGGACAAGGAGA | 60 | NM_008302.3 |
|  | Reversal: CGTCGGTTAGTGGAATCTTCATG |  |  |
| *Ifng* | Forward: CAACAGCAAGGCGAAAAAGG | 58 | NM_008337.4 |
|  | Reversal: GGACCACTCGGATGAGCTCA |  |  |
| *Il4* | Forward: GTCACAGGAGAAGGGACGCCAT | 59 | NM_021283.2 |
|  | Reversal: AGCCCTACAGACGAGCTCACTC |  |  |
| *Il6* | Forward: CCGGAGAGGAGACTTCACAG | 59 | NM_031168.2 |
|  | Reversal: TCCACGATTTCCCAGAGAAC |  |  |
| *Il7* | Forward: CGCAGACCATGTTCCATGT | 58 | NM_008371.5 |
|  | Reversal: TCTTTAATGTGGCACTCAGATGAT |  |  |
| *Il10* | Forward: AGGACTTTAAGGGTTACTTGGGTT | 59 | NM_010548.2 |
|  | Reversal: GCTCCACTGCCTTGCTCTTATT |  |  |
| *Il12a* | Forward: GTGTCTTAGCCAGTCCCGAA | 59 | NM_008351.3 |
|  | Reversal: TTCAAGTCCTCATAGATGCTACCAA |  |  |
| *Il17a* | Forward: GGACTCTCCACCGCAATGAA | 59 | NM_010552.3 |
|  | Reversal: CATGTGGTGGTCCAGCTTTC |  |  |
| *Inos* | Forward: CTCGGAGGTTCACCTCACTGT | 59 | NM_010927.4 |
|  | Reversal: GCTGGAAGCCACTGACACTT |  |  |
| *Tgfb1* | Forward: ATAGCAACAATTCCTGGCGT | 59 | NM_011577.2 |
|  | Reversal: CCTGTATTCCGTCTCCTTGG |  |  |
| *Tnfa* | Forward: GAAGTTCCCAAATGGCCTCC | 58 | NM_013693.3 |
|  | Reversal: CACTTGGTGGTTTGCTACGA |  |  |
